# The effects of a genome-wide supported variant in the *CACNA1C* gene on cortical morphology in schizophrenia patients and healthy subjects

**DOI:** 10.1038/srep34298

**Published:** 2016-09-29

**Authors:** Fanfan Zheng, Yue Cui, Hao Yan, Bing Liu, Tianzi Jiang

**Affiliations:** 1 Brainnetome Center, Institute of Automation, Chinese Academy of Sciences, Beijing, China; 2 National Laboratory of Pattern Recognition, Institute of Automation, Chinese Academy of Sciences, Beijing, China; 3 Institute of Mental Health, The Sixth Hospital, Peking University, Beijing, China; 4 Key Laboratory of Mental Health, Ministry of Health & National Clinical Research Center for Mental Disorders (Peking University), Beijing, China; 5 Queensland Brain Institute, The University of Queensland, Brisbane, QLD, Australia; 6 Key Laboratory for NeuroInformation of Ministry of Education, School of Life Science and Technology, University of Electronic Science and Technology of China, Chengdu, China

## Abstract

Schizophrenia is a highly heritable disorder with multiple susceptibility genes. Previously, we identified *CACNA1C* rs2007044 as a new risk locus for schizophrenia, with the minor allele G as risk allele. This association was recently validated by a powerful genome-wide association study. However, the underlying neural mechanisms remain unclear. Therefore, we tested whether the risk allele has an influence on cortical surface area and thickness in a sample of schizophrenia patients and healthy controls. We found significant genotype by diagnosis interactions on cortical surface area, but not thickness, in the right dorsolateral prefrontal cortex and the left superior parietal cortex, both of which are key components of the central executive network. Moreover, the surface areas of both regions were inversely correlated with PANSS negative scores in AA homogeneous patients but not in G-carriers. This is the first study to describe the influence of the new genome-wide supported schizophrenia risk variant on cortical morphology. Our data revealed a significant genetic effect of cortical surface area in pivotal brain regions, which have been implicated in the pathophysiology of schizophrenia, possibly via their involvement in cognitive functions. These results yield new insights into the potential neural mechanisms linking *CACNA1C* to the risk of schizophrenia.

Schizophrenia is a complex psychiatric disorder characterized by various clinical symptoms, including positive symptoms, negative symptoms and cognitive impairments. The negative symptoms, which were mainly described as lack of motivation and social interactions, flat expressions of affective experience and responsiveness, poverty of speech, and slowed movement, contribute more to poor functional outcomes and tend to persist longer and be more difficult to treat than positive symptoms^1^. Family and twin studies have suggested that, of the major psychiatric disorders, schizophrenia has one of the highest estimates of heritability, ranging from 60% to 90%^2^. However, the genetic mechanisms underlying the numerous findings from association studies remain largely unknown^3^. Given the phenotypic heterogeneity of schizophrenia patients, neurocognitive deficits and neuroimaging-based phenotypes have been introduced into molecular genetic analysis as endophenotypes to reduce the potential confounding effect of disease phenotypic heterogeneity^4^,^5^.

One of the well-recognized susceptibility genes for schizophrenia is *CACNA1C*, which encodes the alpha-1c subunit of the L-type voltage-gated calcium channel^6^,^7^,^8^,^9^. Calcium channels are essential elements for converting electrical activity into biochemical events. Calcium enters the cell through these channels and regulates ion pumps, enzymes, and components of the cytoskeleton^10^,^11^. Therefore, variations at *CACNA1C* could affect signal transduction and cytoskeleton plasticity and thus lead to aberrant brain structure and function, which may contribute to the etiology of schizophrenia. In a previous study, our group identified a new risk locus in *CACNA1C*, rs2007044, as being associated with schizophrenia^8^. This association was further confirmed by the recent powerful genome-wide association study (GWAS) conducted by the Schizophrenia Working Group of the Psychiatric Genomics Consortium, which ranked it 4^th^ in the 108 identified schizophrenia-associated genetic loci^9^. As a next step, imaging studies are necessary to test whether variation at rs2007044 could affect brain structure and function.

In recent years, imaging genetics has emerged as a promising approach that enables the identification and characterization of the influence of genetic variants on brain structure and function^12^. Therefore, we applied an imaging genetics approach to investigate the potential effect of rs2007044 on cortical morphology. We measured two parameters of cortical morphology, surface area and thickness, as alterations in them have repeatedly been observed in schizophrenia and their unaffected relatives^13^,^14^,^15^,^16^. Moreover, both morphological measures are highly heritable^17^,^18^. Taken together, the characteristics mentioned above make cortical surface area and thickness suitable intermediate phenotypes for our imaging genetics approach, based on the standards proposed by Gottesman and Gould^19^.

According to previous studies, the minor allele G of rs2007044 has been shown to confer a risk effect that increases disease susceptibility, whereas allele A is likely to confer a protective effect^8^,^9^. Therefore, we hypothesized that carriers of allele G would exhibit aberrant cortical surface area and/or cortical thickness in comparison to homozygous AA individuals.

## Results

### Demographic and genetic characteristics

The demographic and clinical characteristics of the subjects are summarized in Table 1. No significant differences in age, gender, and educational years were identified between the patients with schizophrenia and the healthy controls. In addition, no significant differences were detected in these variables between the genotypes in either of the two diagnostic groups (Table 1). The genotype distributions for patients and controls were in Hardy-Weinberg equilibrium (*p* > 0.05).

### *CACNA1C* rs2007044 and cortical morphology

**Figure 1:**
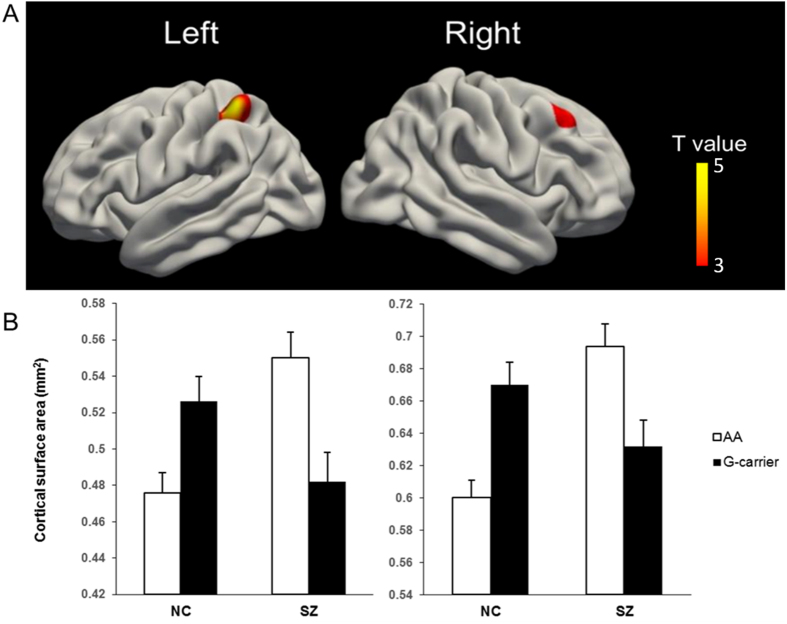
The upper maps illustrate the brain regions which showed significant genotype by diagnosis interaction effects after using the random field theory (RFT)-based multiple comparison correction with *p* < 0.05 across the whole brain. The histograms show the cortical surface areas (Mean ± S.E.) of the corresponding brain regions above them. The Y axis indicates the mean surface area of all the vertices in the cluster. NC, Normal healthy controls; SZ, Schizophrenia patients.

We found significant interactions between genotype and diagnosis group for the cortical surface area in the right dorsolateral prefrontal cortex (DLPFC, BA9, *p* = 0.042 after RFT correction) and the left superior parietal cortex (SPC, BA7, *p* = 0.011 after RFT correction). Both regions exhibited the same pattern of interaction effects, with patients who were G carriers showing reduced cortical surface area compared with the AA homozygotes in contrast to the opposite trend in the healthy controls (G carriers having increased cortical surface area compared to AA homozygotes), as shown in Fig. 1. The main effects of neither genotype nor diagnosis could survive the RFT correction. For cortical thickness, no significant effect of the risk variant was found in either the schizophrenia patients or the healthy controls.

### Cortical surface area and PANSS scores

**Figure 2:**
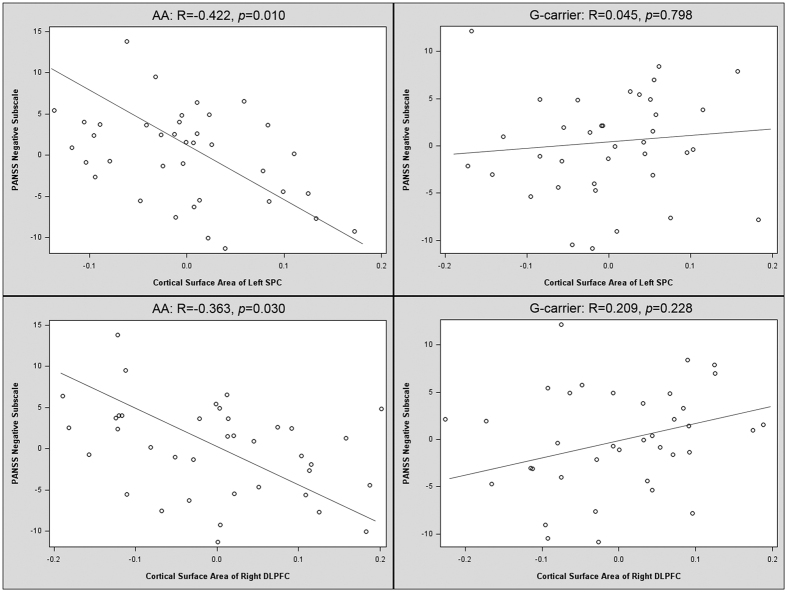
Significant correlations between the cortical surface area and negative symptom severity for AA homogeneous patients but not G-carriers. Plotted values are residuals after adjustment for covariates.

In patients who were homogeneous non-risk A allele individuals, the cortical surface areas of the right DLPFC (R = −0.363, *p* = 0.030) and the left SPC (R = −0.422, *p* = 0.010) were each inversely correlated with the PANSS negative scores. However, no such correlation was observed in the risk G allele carriers (Fig. 2).

## Discussion

The present study employed an imaging genetics strategy to investigate the effect of *CACNA1C* rs2007044, a schizophrenia susceptibility SNP first identified by our previous study and then confirmed by a recent GWAS, on cortical surface area and thickness. Our results showed significant genotype by diagnosis interactions for the cortical surface area in the right DLPFC and the left SPC. There was no significant difference of cortical surface area between the schizophrenia patients and the healthy controls, which was consistent with findings of previous studies^20^,^21^. Moreover, the cortical surface areas in both regions were inversely correlated with the PANSS negative scores in AA homogeneous patients but not the risk G-allele carriers. These findings confirmed our hypothesis that variations at rs2007044 impact cortical morphology and provide important implications about individual differences in the genetic association of rs2007044 with cortical surface area and the risk of developing schizophrenia.

One of our main findings was that the cortical surface area showed significant genotype by diagnosis group interaction effects in the right DLPFC and the left SPC. Both of these regions have previously been implicated in the pathophysiology of schizophrenia^22^. Prefrontal dysfunction is thought to be one of the major constituents of aberrant cognitive control in schizophrenia, with the right DLPFC being one of the most consistently identified regions^22^. Overactivation of the right DLPFC during a working memory task has been considered to be a manifestation of prefrontal inefficiency^23^, and has repeatedly been observed in schizophrenia patients and healthy individuals with high genetic risks^23^,^24^,^25^,^26^. By using functional MRI, a previous study reported that *CACNA1C* rs1006737 genotype was associated with alteration of brain activation in the right DLPFC during working memory tasks^25^. Although the investigated SNP was different, this finding was compatible with our results and supported that *CACNA1C* genotypes might modulate working memory function through their impacts on the right DLPFC. The SPC is also known to be a crucial structure for the manipulation and rearrangement of information during working memory^27^. Because deficits in working memory have long been recognized as one of the major pathophysiological features of schizophrenia patients^28^, the alteration in the cortical surface of the DLPFC and SPC might be the potential effects through which rs2007044 contributes to the cognitive deficits of schizophrenia. This is consistent with previous findings indicating that both prefrontal and superior parietal dysfunction are major elements in the context of the disturbed cognitive control and associated altered working memory deficits in schizophrenia^22^. Nevertheless, our findings of laterality effects, which possibly stem from the functional asymmetry of the cerebral hemispheres, deserve to be mentioned. It has been argued that, slightly unlike the left DLPFC, which mainly active during longer memory delays, the right DLPFC is reported to maintain the active representation of stimuli over short periods of time during working memory execution^29^. Apart from working memory, hemispheric differences of the DLPFC have also been observed in the context of impulse control and decision making^30^,^31^. Similarly, functional asymmetry of the SPC for working memory has also been reported, with the findings that the left SPC contributed more to executive function and the right SPC was more strongly involved in short-term storage^32^. Thus, we may speculate that rs2007044 exerts its effects via asymmetric brain regions and influences relevant aspects of brain functions. Altogether, our findings lend support to the view that *CACNA1C* might be involved in impaired cognitive functions, especially working memory, in schizophrenia.

Besides, it is noteworthy that, while patients who were G carriers showing reduced cortical surface area compared with the AA homozygotes, the healthy G carriers displayed increased cortical surface area in both the right DLPFC and the left SPC. In view of previous studies indicating that both schizophrenia patients and individuals at greater risk for schizophrenia exhibited relatively more inefficient information processing during working memory performance^23^,^24^,^25^,^26^, it is possible that the expanded cortical surface area observed in healthy risk G-allele carriers may represent a compensational mechanism to maintain sufficient information processing and achieve normal behavior. An alternative explanation for this opposite trend may be related to the complexity of interactions between genotype and environment, genotype and illness, and even across genotypes. Similar opposite trends on brain structure and function have been reported by previous studies^33^,^34^, and the precise mechanisms still await elucidation.

Another interesting finding of our current study was that the surface area of the individual patients was associated with their PANSS negative score in a totally different way depending on the rs2007044 genotype. In homogeneous A carriers, who may have a lower genetic risk for schizophrenia, the surface areas in both clusters were inversely correlated with the patients’ PANSS negative scores. Such correlations indicate that individuals with a smaller cortical surface area may have a higher PANSS negative score, which can be equated to more severe negative symptoms. These results corroborate a prior study reporting correlations between right frontal and left superior parietal reductions and negative symptoms^35^. Currently, the relationship between cognitive functions and negative symptoms is still under debate. Several studies have suggested that negative symptoms are related to cognitive performance^36^,^37^,^38^, while other studies indicated that negative symptoms did not appear to be related to neurocognitive dysfunction^39^,^40^. Our findings of a correlation between negative symptoms and the cortical surface area of pivotal brain regions involved in cognitive functions may partially support a potential relationship between cognitive function and negative symptoms. This is compatible with meta-analyses that reported small to moderate correlations between cognitive performance and negative symptoms^41^,^42^. However, this inverse correlation disappeared in the risk G allele carriers. This disappearance might be due to the significantly decreased cortical surface area associated with the genetic effect of the risk G allele in the patients.

This is the first study to employ an imaging genetics approach to investigate the effect of *CACNA1C* rs2007044 on cortical morphology. Previous research studies on *CACNA1C* primarily focused on the genetic effect of another SNP, rs1006737, which GWASs found to be associated with schizophrenia in European populations^6^,^7^, but which has a much lower minor allele frequency (MAF) in East Asian populations (http://hapmap.ncbi.nlm.nih.gov/). Unlike rs1006737, rs2007044 has approximate MAFs between different ethnic populations. Moreover, the results of the effects of rs1006737 on cortical morphology were inconsistent, with some reporting changes in regional cortical volume^43^,^44^ while others finding no difference in either the regional cortical volume or the subcortical grey matter volume^45^,^46^. It is worth noting that cortical volume comprises both surface area and thickness, and previous studies suggested that these two components of cortical volume are genetically and phenotypically independent^17^,^47^. Therefore, we separately explored the effect of rs2007044 on both the cortical surface area and thickness. Interestingly, we found significant genetic effects only for the cortical surface area but not for the cortical thickness. This observation provides further support for accumulating evidence that specific neuroimaging features can be genetically and phenotypically independent^17^,^47^. According to the radial unit hypothesis, cortical surface area and cortical thickness are related to the number of cellular columns and the number of cells in a column, respectively^48^. In addition, evidence from a biological study suggested that cortical surface area is determined by intermediate progenitor cells whereas cortical thickness is determined by radial progenitor cells^47^. Moreover, recent evidence from a twin study indicated that the cortical surface area of the mature adult brain seems to be impacted by genes during both early and later development of the brain, with subsequent effects of processes such as synaptogenesis and dendritic arborization^17^. Taken all those findings into account, our present data suggest that *CACNA1C* could potentially impact the biological processes more relevant to cortical surface area and thus contribute to the pathophysiology of schizophrenia. In fact, voltage-gated calcium channels have already been shown to be involved in neuronal development during both early development and adulthood^49^. *CACNA1C* has long been implicated in the development of brain structure and function^50^. Coding for the major L-type voltage-gated calcium channel, *CACNA1C* plays crucial roles in regulating the activity-dependent influx of calcium, which in turn modulates the downstream signal transduction, including the regulation of some calcium-dependent genes such as brain-derived neurotrophic factor (BDNF) and B-cell lymphoma 2 (BCL-2), both of which impact brain development and cortical structure, as indicated by solid biological evidence^51^,^52^,^53^,^54^. Due to our previous finding that variations at rs2007044 may change the binding affinity of transcription factor and alter the promoter activity of *CACNA1C*^8^, the observed effects of rs2007044 on the cortical surface area may, at least in part, be due to changes in the *CACNA1C* expression level along with its subsequent downstream effects. Although no direct evidence about the biological function of rs2007044 has been identified yet, our current findings yield new insights into understanding the biological role of *CACNA1C* during cortical development and deserve further exploration.

The results of our study are intriguing, but several issues should be noted. First of all, since this is the first study concerning the genetic effects of rs2007044, replications are needed to validate our findings. Second, the potential cellular and molecular biological mechanisms underlying our findings are beyond the resolution of imaging study. Future studies should pay more attention to the function of *CACNA1C* at the cellular and molecular level. With the help of genome-editing technologies, such as TALEN or CRISPR^55^,^56^, direct evidence revealing the biological role of *CACNA1C*, even the effect of rs2007044 in the absence of other genomic variability, could be obtained. Furthermore, because this study lacked longitudinal data, we cannot provide information about differences in psychosis outcomes, which might be affected by the risk variant in association with its possible influence on cortical surface area. Thus, longitudinal studies on this issue are warranted. In addition, although our data implied that variations at rs2007044 may impact cognitive functions, such as working memory, the current study did not collect working memory data and could not investigate the direct link between them. Future studies in this area should also collect task-related data, which may provide more information regarding the genetic effects of rs2007044 with cognitive functions. Last but not least, our study did not specifically exclude subjects who smoked but only required them not to smoke for at least 24 hours before scanning. Although smoking was reported to mainly impact cortical thickness according to previous studies^57^,^58^, our results of genetic effects on cortical surface area should be interpreted with caution.

In conclusion, the present study demonstrated disease-specific genetic effects of *CACNA1C* rs2007044 on the cortical surface area of the DLPFC and SPC. Both of these regions play key roles in high-order cognitive functions and have been previously implicated in the pathophysiology of schizophrenia. Our data may therefore provide new insights into the potential neural mechanism linking rs2007044 to the risk of schizophrenia. The precise mechanisms through which rs2007044 might exert its influence on cortical morphology remain to be elucidated by future studies using animal models or other techniques.

## Methods

### Subjects

We recruited a total of 85 schizophrenia patients and 99 healthy control subjects. All the subjects were Han Chinese from northern China. Consensus diagnoses were made by two experienced senior psychiatrists using the Diagnostic and Statistical Manual of Mental Disorders, 4^th^ edition (DSM-IV) criteria for schizophrenia, based on the Structured Clinical interview for DSM-IV-TR Axis I Disorders (SCID-I, patient edition). Patients with any other neurologic disorder, a history of severe medical illness, drug and alcohol dependence, pregnancy, or treatment with electroconvulsive therapy within the past 6 months and those with a diagnosis of any other Axis I disorder were excluded. The symptom severity of all the patients was assessed by trained and experienced psychiatrists using the Positive and Negative Syndrome Scale (PANSS) within one week prior to MRI scanning. Healthy controls were recruited from the community and screened using the SCID-I (non-patient edition). Individuals with any history of mental and/or neurological disorder were excluded. Written informed consent was obtained from all patients and their legal guardians (i.e., their parents) and all healthy controls. The study was approved by the Medical Research Ethics Committee of the Institute of Mental Health, Peking University. All experiments were performed in accordance with approved guidelines and regulations.

### Genotyping

Peripheral blood samples were collected from all subjects. Genomic DNA was extracted from the blood using a Qiagen QIAamp DNA Mini Kit. Rs2007044 was genotyped using a TaqMan SNP genotyping assay on an ABI PRISM 7900 Sequence Detection Systems (Applied Biosystems, Foster City, CA), using our previously described method^8^. For quality control purposes, all the genotypes were blind to the case or control status during the genotyping process. We repeated the genotyping assay for ten percent of the samples and found that the results were 100% concordant.

### Structural MRI acquisition

The MRI scanning was carried out on a Siemens 3.0 Tesla Trio MR scanner at the Third Hospital, Peking University. Three-dimensional T1-weighted images were acquired in a sagittal orientation employing a 3D-MPRAGE sequence with the following parameters: time repetition (TR) = 2350 ms, time echo (TE) = 3.44 ms, flip angle = 7°, matrix size = 256 × 256, field of view (FOV) = 256 × 256 mm^2^, 192 sagittal slices, slice thickness/gap = 1.0/0 mm, acquisition voxel size = 1 × 1 × 1 mm^3^. Foam pads were used to minimize head motion. A quality control to exclude motion artifacts was carried out independently by 2 researchers. Because of motion artifacts and missing data, 75 patients and 97 controls remained in the subsequent analysis. All the remained patients are paranoid schizophrenia patients.

### Calculation of cortical morphology

We used the FreeSurfer software package, version 5.3.0 (http://surfer.nmr.mgh.harvard.edu) for image processing and for thickness and surface area analyses, using our previously reported process^59^. In brief, the stripped, intensity-corrected, subdivided volume was segmented to classify the white matter and to approximate the gray-white matter boundary for each cortical hemisphere. From this, a topologically correct, gray-white matter boundary (white) surface triangulation was generated^60^,^61^. Subsequently, a pial surface was generated using a deformable surface algorithm. The surface reconstruction results for each subject were visually checked before further analysis. We then obtained a vertex-wise cortical thickness map by calculating the shortest distance between the pial and white surfaces^62^,^63^. Vertexwise estimates of the surface area were calculated by assigning one-third of the area of each triangle to each of its vertices. For comparison, the reconstructed cortical surfaces for each individual were aligned to an average template by using a surface-based registration algorithm. The thickness and area maps were resampled and smoothed with a Gaussian kernel of full width at half maximum of 20 mm.

### Statistical analyses

IBM SPSS 22.0 was used for descriptive statistical analysis. Independent-sample *t* tests and *chi* square tests were used for continuous and discrete variable comparisons. Statistically significant differences were defined as *p* < 0.05.

Vertex-by-vertex analyses of cortical thickness and surface area were performed using the SurfStat package (http://www.math.mcgill.ca/keith/surfstat/). The patients and healthy controls were separately divided into two subgroups, AA and (AG + GG), depending on their rs2007044 genotypes. A general linear model (GLM) was used to explore the differences in the cortical thickness/surface area between the genotype subgroups, with age and gender as covariates. We used the random field theory (RFT)-based multiple comparison correction with *p* < 0.05 across the whole brain to reduce the possibility of obtaining false positives. In the schizophrenia patients’ group, a partial correlation (two-tailed) was also used to examine the relationship between the cortical parameters and the PANSS positive/negative score after controlling for age and gender.

## Additional Information

**How to cite this article**: Zheng, F. *et al*. The effects of a genome-wide supported variant in the *CACNA1C* gene on cortical morphology in schizophrenia patients and healthy subjects. *Sci. Rep.* **6**, 34298; doi: 10.1038/srep34298 (2016).

## Figures and Tables

**Table 1:** Demographic and clinical characteristics of schizophrenia patients and healthy controls.

Variable	Schizophrenia patients				Healthy controls				
	Total	AA	G-carrier	*p* value	Total	AA	G-carrier	*p* value	*p* value
Gender (Male/Female)	46/29	24/14	22/15	0.742	51/46	27/27	24/19	0.569	0.251
Age in years	27.59 (7.00)	27.72 (6.70)	27.45(7.39)	0.886	25.73 (5.41)	25.69(5.20)	25.78(5.72)	0.937	0.059
Education in years	13.75 (2.91)	13.95 (2.69)	13.54(3.14)	0.548	13.60 (3.39)	13.52 (3.74)	13.71 (2.91)	0.780	0.768
PANSS positive score	23.62 (4.40)	23.76 (4.54)	23.81(4.43)	0.963	n.a.	n.a.	n.a.	n.a.	n.a.
PANSS negative score	18.68 (5.96)	17.87 (5.79)	18.57(5.48)	0.593	n.a.	n.a.	n.a.	n.a.	n.a.

PANSS, Positive and Negative Syndrome Scale; n.a.: not applicable.

Data are given as mean (standard deviation), unless otherwise indicated.
